# Fucoidan as a renal protectant: mechanistic insights and therapeutic implications of endothelial glycocalyx targeting

**DOI:** 10.3389/fphar.2026.1749109

**Published:** 2026-01-16

**Authors:** Ping Xin, Chengqiao Ge, Yufan Tang, Yifan He, Bin Fu

**Affiliations:** 1 College of Traditional Chinese Medicine, Tianjin University of Traditional Chinese Medicine, Tianjin, China; 2 The Second Affiliated Hospital of Tianjin University of Traditional Chinese Medicine, Tianjin, China

**Keywords:** charge barrier, chronic kidney disease, endothelial glycocalyx, fucoidan, glycosaminoglycan, heparan sulfate, proteinuria

## Abstract

The progression of chronic kidney disease (CKD) is closely associated with damage to the endothelial glycocalyx (eGC) of the renal microvasculature. The eGC, particularly its heparan sulfate (HS) components, is crucial for maintaining the charge-selective barrier and microenvironmental homeostasis. Modern pharmacological investigations of marine brown algae (e.g., *Saccharina japonica*), traditionally used in medicine for conditions such as “edema,” reveal that their principal active component, fucoidan, is a sulfated polysaccharide with marked physicochemical similarities to endogenous HS. This review systematically posits that the core mechanism underlying the nephroprotective effects of fucoidan, as a natural product, lies in its direct targeting and repair of the damaged eGC. Through a systematic literature search up to November 2025, this review elucidates that fucoidan, especially its low-molecular-weight fractions, can consolidate and reconstitute the glycocalyx structure via dynamic integration, competitive substitution, and activation of intracellular signaling pathways. This central action not only directly restores the renal charge barrier and reduces proteinuria but also, by stabilizing endothelial function, systemically inhibits the inflammation and fibrosis cascades triggered by glycocalyx injury. The efficacy of fucoidan in diverse preclinical models, coupled with clinical trial evidence for fucoidan-based drugs in human CKD patients, collectively supports the validity of a glycocalyx-targeted therapeutic strategy. We conclude that fucoidan represents a natural product derived from traditional wisdom, with a defined molecular mechanism and translational potential, offering a promising complementary strategy for the comprehensive management of CKD.

## 1 Introduction

Chronic kidney disease (CKD) poses a formidable global public health challenge, affecting over 700 million individuals (Francis et al., 2024). Its progression is characterized by persistent inflammation, oxidative stress, and renal fibrosis (Rayego-Mateos and Valdivielso, 2020). Current standard therapies, predominantly represented by renin-angiotensin system inhibitors (Kalantar-Zadeh et al., 2021; Kidney Disease: Improving Global Outcomes CKD Work Group 2024), often inadequately halt renal fibrosis, underscoring an urgent need for novel strategies. Recent research focus has shifted towards microvascular endothelial dysfunction and its luminal surface layer, the glycocalyx (Ying et al., 2023b). The endothelial glycocalyx is a complex mesh-like structure composed primarily of glycosaminoglycans (e.g., heparan sulfate, HS), proteoglycans, and glycoproteins (Ricard-Blum et al., 2024). In the kidney, particularly within glomerular capillaries, an intact glycocalyx serves as the anatomical basis for the charge-selective barrier, preventing the filtration of anionic proteins like albumin (Masola et al., 2021; Foote et al., 2022), and precisely regulates vascular permeability, inflammatory cell adhesion, and growth factor activity (Dancy et al., 2024; Wong et al., 2024). In CKD, various factors (e.g., hyperglycemia, hypertension, uremic toxins) increase reactive oxygen species (ROS) and inflammatory cytokines, subsequently activating degradative enzymes like heparanase, thereby disrupting the eGC “synthesis-degradation” balance. This leads to glycocalyx shedding, HS loss, and charge barrier collapse, manifesting as proteinuria and further activating potent inflammatory and fibrotic signaling networks. Consequently, repairing the endothelial glycocalyx has emerged as a promising new strategy for renal protection (Ying et al., 2023a; Huang et al., 2020).

Concurrently, traditional medical wisdom provides clues for discovering such therapies (Chen et al., 2023; Cheng and Cheng, 2019). Marine medicinal algae, notably “Haizao” (*Sargassum*) and “Kunbu” (*Laminaria*), have a long history of use in China for symptoms like “edema” (Wang, 2011; Chen, 2016; Huang et al., 2015), corresponding to the clinical manifestations of modern kidney diseases (Ren and Wu, 2025). Modern pharmacological studies indicate that fucoidan is a major active constituent common to these herbs, characterized as a sulfated polysaccharide (Zahan et al., 2022; An et al., 2022). Based on its significant protective effects in various renal injury models (Gao et al., 2022; Yu et al., 2020), we propose the core hypothesis of this review: the protective effect of fucoidan against CKD primarily stems from its physicochemical similarity to endogenous HS (Dagälv et al., 2022), enabling it to directly target, integrate into, and repair the damaged renal endothelial glycocalyx, thereby conferring broad renal benefits through the restoration of endothelial homeostasis. This article will systematically integrate existing evidence to elaborate on this mechanism and discuss its clinical implications and future directions. The detailed literature search strategy, including inclusion and exclusion criteria, is provided in the Supplementary Material.

## 2 Chemical structure, sources, and structure-activity relationship of fucoidan

Fucoidan is a natural sulfated polysaccharide extracted from marine brown algae (Ale et al., 2011). Common sources include various brown algae listed in the Chinese Pharmacopoeia (Chinese Pharmacopoeia Commission, 2020), such as *Sargassum pallidum* (Turner) C. Agardh (Zhang et al., 2025), *Sargassum fusiforme* (Harvey) Setchell (Lei et al., 2025), *Saccharina japonica* (Areschoug) Lane, Mayes, Druehl and Saunders (also known as *Laminaria japonica*) (Luan et al., 2021), and *Ecklonia kurome Okamura* (Zha et al., 2015). Its chemical structure is based on a backbone of α-L-fucopyranose residues predominantly linked via α-(1→3) and/or α-(1→4) glycosidic bonds, with sulfate ester groups commonly substituted at the C-2 and/or C-4 positions (Cui et al., 2022).

The biological activity of fucoidan is highly structure-dependent (Li et al., 2020). Its molecular weight (MW) distribution is broad and influenced by algal source and extraction processes. For instance, fucoidan from Saccharina japonica, most commonly used for renal protection, typically has an MW ranging from approximately 7 kDa (Wang et al., 2020) to 2,000 kDa (He et al., 2007). Degradation to low-molecular-weight fucoidan (LMWF, typically <10 kDa) (Xu et al., 2016; Gao et al., 2023) or fucoidan oligosaccharides (typically ∼800 Da) (Yu et al., 2020) generally confers superior renoprotective activity. This is attributed to: (i) improved solubility and reduced steric hindrance, facilitating tissue diffusion and access to cell-surface targets; (ii) enhanced potential for transmembrane transport and cellular internalization, allowing uptake by endothelial cells to modulate intracellular signaling pathways (detailed in Section 3.3); and (iii) favorable pharmacokinetic properties, including better oral absorption and more pronounced renal distribution (detailed in Section 3.4). Sulfate groups confer a polyanionic character to fucoidan. Its degree of sulfation (sulfate content typically ranging from 6.01% to 38.3%) (Hamouda et al., 2025) directly correlates with its negative charge density. High sulfation effectively mimics the electrostatic properties of endogenous HS, providing the chemical basis for strong electrostatic interactions with HS-binding proteins, such as growth factors, enzymes, and the heparin-binding domains (HBDs) on glycocalyx core proteins (Bilan et al., 2017; Yue et al., 2025). This structural similarity is the prerequisite for its ability to target and integrate into damaged glycocalyx sites. Furthermore, fucoidans from different sources may contain other monosaccharides (e.g., galactose, xylose, glucuronic acid) and vary in chain branching and linkage patterns, potentially affecting their binding specificity and affinity for particular protein targets.

## 3 Direct protection and repair of the endothelial glycocalyx by fucoidan

### 3.1 Role of the endothelial glycocalyx in renal physiology and pathology

The endothelial glycocalyx of glomerular capillaries, especially its abundant HS side chains (Masola et al., 2021), forms the primary basis of the glomerular charge barrier, electrostatically repelling anionic proteins like albumin (Prasad et al., 2025). It also functions as a dynamic signaling platform that precisely regulates vascular tone, inflammatory responses, and tissue repair by binding and modulating growth factors, enzymes, and chemokines (Dancy et al., 2024; Wong et al., 2024). During CKD progression, multiple pathological factors (e.g., hyperglycemia, hypertension, uremic toxins) synergistically induce excessive reactive oxygen species (ROS) production and promote the release of inflammatory cytokines like tumor necrosis factor-α (TNF-α) and interleukin-1β (IL-1β) (Lepedda et al., 2017). This upregulates the expression and activity of degradative enzymes, including heparanase (HPSE) and matrix metalloproteinases (MMPs) (Buijsers et al., 2023; Haddad et al., 2015; Patterson et al., 2022). HPSE, the key rate-limiting enzyme cleaving HS chains, is central to enzymatic glycocalyx degradation, directly causing extensive shedding, significant HS loss, and charge barrier collapse. Such structural damage, confirmed by transmission electron microscopy in CKD animal models and human kidney specimens, is evidenced by a marked reduction in the thickness and density of the peritubular capillary glycocalyx (Ermert et al., 2023). Elevated levels of circulating glycocalyx degradation products (e.g., soluble syndecan-1, hyaluronan) (Yuan et al., 2022) correlate positively with clinical disease severity. In clinical studies, these markers are associated with increased urinary albumin-to-creatinine ratio (UACR), elevated systemic inflammatory markers (e.g., IL-6, TNF-α), faster decline in estimated glomerular filtration rate (eGFR), and increased cardiovascular event risk (Kim et al., 2017).

Among various pathogenic factors, protein-bound uremic toxins, notably indoxyl sulfate (IS), are considered key drivers of glycocalyx injury due to their significant accumulation in renal impairment and direct attack on endothelial cells. The molecular mechanism is increasingly elucidated: IS activates endothelial NADPH oxidase (particularly the Nox4 subunit), induces mitochondrial dysfunction and excessive ROS production, and subsequently activates the ERK/p38 mitogen-activated protein kinase (MAPK)–nuclear factor-kappa B (NF-κB) signaling pathway. This upregulates the expression of pro-inflammatory factors like monocyte chemoattractant protein-1 (MCP-1) and intercellular adhesion molecule-1 (ICAM-1) and promotes HPSE release, thereby accelerating HS degradation (Masai et al., 2010).

Therefore, endothelial glycocalyx injury is both an early structural marker of microvascular pathology in CKD and a critical pathological link driving proteinuria and subsequent inflammatory-fibrotic cascades. Dynamic changes in circulating glycocalyx components not only reflect the extent of endothelial injury but also offer a potential biomarker window for non-invasive monitoring of disease activity and endothelial health.

### 3.2 Immediate repair of the charge barrier

Facing the exposed, positively charged HBDs of core proteins (e.g., syndecan-1) following HS loss (Jiang et al., 2021; Diab et al., 2024), fucoidan executes immediate, dynamic integration based on its striking physicochemical similarity to endogenous HS (Kuo et al., 2019). The essence of this action is structure-based competitive functional substitution. The high-density sulfate groups on the fucoidan backbone render it a strong polyanion. Through precise electrostatic interactions (notably salt bridge formation with arginine residues in HBDs) and hydrogen bonding networks (Li et al., 2025), fucoidan reversibly yet with high affinity anchors at injury sites. Biophysical studies support the existence of such specific interactions (Kabedev and Lobaskin, 2022). Additionally, specific molecular recognition mechanisms (e.g., CH-π interactions) between fucose units and proteins may further facilitate this integration (Zhu et al., 2024). This immediate competitive integration rapidly restores local glycocalyx negative charge density and molecular sieve properties. *In vitro* experiments and animal models (Xu et al., 2016; Xue et al., 2025; Yu et al., 2020) confirm that low-molecular-weight fucoidan (LMWF) or oligo-fucoidan significantly reduces endothelial albumin permeability induced by inflammatory factors, with superior effects compared to high-MW fucoidan (Tan et al., 2020). This mechanism was directly validated in the passive Heymann nephritis model, a model of secondary glycocalyx injury (Zhang et al., 2005), where proteinuria was linked to HS reduction in the glomerular basement membrane (GBM) attributed to complement-mediated cleavage, ultimately increasing GBM permeability to macromolecules. That study directly observed that fucoidan restored glomerular charge selectivity, corroborating its core mechanism of mimicking HS and competitively integrating into damaged sites to restore filtration barrier function.

### 3.3 PI3K/Akt and ERK/MAPK signaling pathway-mediated active glycocalyx repair

Fucoidan’s action on the eGC extends beyond mere physical substitution, involving bidirectional active regulation of its metabolism, primarily manifesting in inhibiting degradation and promoting synthesis. Regarding degradation inhibition, fucoidan, as an HS structural analog, can competitively inhibit the activity of the key degrading enzyme HPSE. Its inhibitory potency correlates positively with the degree of sulfation, with fully sulfated derivatives showing the strongest inhibitory capacity (Sugimoto et al., 2025; Mo et al., 2025). Concerning synthesis promotion and repair, fucoidan activates specific intracellular signaling pathways in endothelial cells, mobilizing cellular regeneration programs. Studies show that fucoidan activates pro-survival and anabolic signaling pathways such as phosphoinositide 3-kinase (PI3K)/protein kinase B (Akt) and extracellular signal-regulated kinase (ERK)/MAPK (Regier et al., 2022). Pathway activation stimulates the Golgi vesicular system to transport pre-synthesized glycocalyx components (e.g., syndecan-1) to the cell surface for rapid replenishment (Regier et al., 2022). It may also promote the transcription of glycocalyx synthesis-related genes (e.g., HS synthesis enzymes EXT1, EXT2) (Haddad et al., 2015), supporting *de novo* synthesis and long-term reconstruction. Furthermore, antioxidant pathways (e.g., nuclear factor erythroid 2–related factor 2, Nrf2) activated by fucoidan upregulate cytoprotective genes like heme oxygenase-1 (HO-1), providing a favorable microenvironment for glycocalyx synthesis by mitigating oxidative stress (Yu et al., 2020). Thus, fucoidan achieves multi-level, active repair of glycocalyx homeostasis by coordinately regulating synthesis-promoting and degradation-inhibiting genes.

### 3.4 Pharmacokinetics, biodistribution, and administration routes

Effective delivery of fucoidan, particularly LMWF, to renal targets is a prerequisite for its renoprotective action. Pharmacokinetic studies support its renal tropism. Animal experiments indicate that after oral or intravenous administration, LMWF accumulates more rapidly and reaches higher concentrations in the kidneys compared to organs like the heart and brain (Wang, 2014; Tan, 2020). Although oral bioavailability reports vary, it is generally considered low (e.g., 2.67%, time to peak ∼2 h, plasma concentration ∼7.33 μg/mL) (Chu, 2017). This limited absorption and subsequent renal distribution are related to LMWF’s improved intestinal absorption via clathrin-mediated endocytosis and its renal filtration and/or binding characteristics (Chu, 2017). Human studies further confirm that low-MW fragments of fucoidan are detectable in urine after oral ingestion by healthy volunteers, providing direct evidence for its absorption and renal delivery in humans (Kadena et al., 2018; Rocha Amorim et al., 2016).

Regarding administration routes, the oral route is primary for approved formulations (e.g., Haikun Shenxi Capsule) (He et al., 2007). Orally administered LMWF is partially absorbed, and its renoprotective effects likely result from a combination of direct systemic effects and indirect effects via gut microbiota modulation (see Section 4). Oral administration offers high compliance, suitable for long-term CKD management. Intravenous administration, while ensuring complete bioavailability and rapid onset—potentially applicable for acute kidney injury—remains investigational, lacking marketed formulations. Preclinical studies suggest a need for cautious safety evaluation, as intravenous administration may significantly prolong clotting time, posing a potential bleeding risk (Sun et al., 2025). Therefore, for the chronic management of CKD, oral administration is currently the more practical and sustainable choice. Future efforts should include head-to-head clinical studies comparing efficacy and safety across routes and exploring nanomaterial-based targeted delivery systems or developing structurally defined, activity-controlled intravenous derivatives (e.g., synthetic fucoidans with selective anticoagulant activity) to overcome intravenous administration bottlenecks.

## 4 Interaction between glycocalyx repair and core CKD pathological networks

**FIGURE 1:**
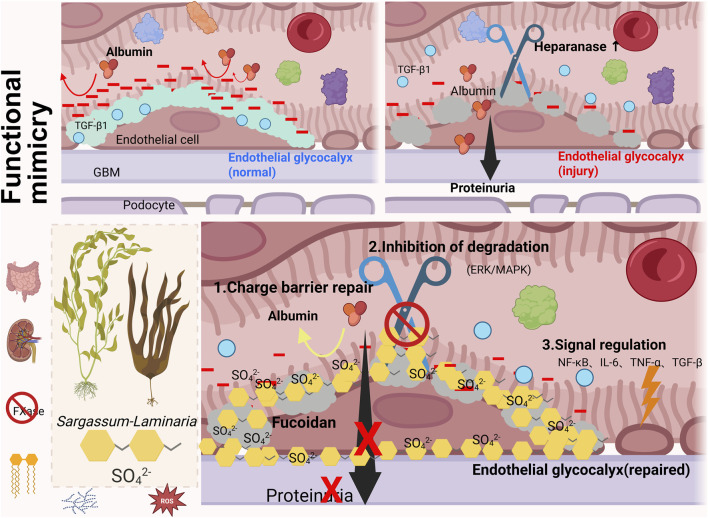
Fucoidan as a Renal Protectant by Targeting the Endothelial Glycocalyx. Note: Light green = normal endothelial glycocalyx, grey = injured endothelial glycocalyx, golden yellow = fucoidan.

By repairing the glycocalyx via the aforementioned mechanisms, fucoidan systematically intervenes in the core pathological mechanisms of CKD—oxidative stress, inflammation, and fibrosis—forming a virtuous cycle (Figure 1).

In countering oxidative stress, fucoidan activates the cytoprotective transcription factor Nrf2, causing its dissociation from Kelch-like ECH-associated protein 1 (Keap1) and nuclear translocation, upregulating the expression of phase II detoxifying enzymes and antioxidant proteins like HO-1 (Yu et al., 2020). This directly scavenges ROS, a key attacker causing glycocalyx degradation, thereby protecting glycocalyx integrity and breaking the vicious cycle of “ROS damages glycocalyx–glycocalyx shedding exacerbates oxidative stress.”

In suppressing the inflammatory cascade, an intact glycocalyx is a physical barrier inhibiting leukocyte adhesion. Fucoidan, by repairing the glycocalyx, directly reduces the initiation of leukocyte rolling and adhesion. Concurrently, it effectively downregulates the production of pro-inflammatory cytokines like TNF-α and IL-6 by inhibiting Toll-like receptor 4 (TLR4) and its downstream NF-κB signaling pathway (Wang et al., 2015; Xu et al., 2016). Notably, NF-κB activation itself upregulates glycocalyx-degrading enzymes like HPSE (Xu et al., 2025); thus, fucoidan’s anti-inflammatory action also indirectly inhibits further glycocalyx destruction.

In curbing the fibrotic process, an intact glycocalyx binds and modulates the bioactivity of pro-fibrotic factors like transforming growth factor-β (TGF-β). Fucoidan-mediated glycocalyx repair restores spatial sequestration and regulation of TGF-β signaling. Moreover, its anti-inflammatory and antioxidant effects collectively create a microenvironment inhibiting myofibroblast activation and excessive extracellular matrix deposition. Studies also indicate that fucoidan can directly downregulate pro-fibrotic signaling pathways such as TGF-β/Smad and Janus kinase 2 (JAK2)/signal transducer and activator of transcription 3 (STAT3) (Chen et al., 2017; Sun et al., 2023).

Additionally, there are other synergistic protective effects. As dietary fiber, fucoidan enriches probiotics (e.g., Akkermansia muciniphila; Wang Z et al., 2025); the short-chain fatty acids (primarily acetate) produced by their fermentation enter circulation and can inhibit renal TLR4 signaling, alleviating inflammation and fibrosis (Zhong et al., 2024; Wang Y. et al., 2025). By activating pathways like Nrf2 and solute carrier family 7 member 11 (SLC7A11)/glutathione peroxidase 4 (GPX4), fucoidan inhibits ROS accumulation and ferroptosis, protecting renal tubular epithelial cells (Chen S. et al., 2025; Cao et al., 2025). Fucoidan improves mitochondrial function (Shi et al., 2025; Veena et al., 2008), activates mitophagy (Gao et al., 2023; Shi et al., 2025), and ameliorates metabolic disturbances like insulin resistance (Xue et al., 2025; Peng et al., 2020), further enhancing renal protection.

## 5 Translational evidence: validation from preclinical models to human efficacy

Numerous preclinical studies have consistently demonstrated the renoprotective effects of fucoidan (especially LMWF) across diverse renal injury models. These models encompass CKD (Wang et al., 2012; Chen et al., 2021; Ma et al., 2022; Li et al., 2017; Katai et al., 2015), renal fibrosis (Sun et al., 2023), nephrotic syndrome (Tan et al., 2020), hyperuricemia (Xue et al., 2024), hyperoxaluria (Veena et al., 2006a; Veena et al., 2006b), metabolic-related injuries like diabetic kidney disease (Cai et al., 2024; Xu et al., 2021; Wang et al., 2021; Wang et al., 2014), and acute or immune-mediated injury models such as ischemia-reperfusion, drug toxicity, and Heymann nephritis (Shu et al., 2021; Jiang et al., 2025; Zhao et al., 2007). Studies commonly report that fucoidan (especially low-MW fractions) significantly reduces proteinuria, improves renal function (e.g., lowering serum creatinine and blood urea nitrogen), alleviates histopathological damage (e.g., glomerulosclerosis, tubular injury, interstitial fibrosis), and downregulates inflammatory and oxidative stress markers.

However, key preclinical studies summarized in Table 1 reveal inherent limitations. First, most animal models (especially acute kidney injury models) fail to fully replicate the complex course and heterogeneity of human CKD. Second, there is significant methodological heterogeneity and underreporting; many studies do not fully characterize the test material’s chemical profile (e.g., MW, degree of sulfation, purity), hindering direct comparison of pharmacological activity and outcomes across fucoidans from different sources. Furthermore, some studies have small sample sizes, and only a few have conducted systematic multi-dose explorations, affecting conclusion reliability and generalizability. These factors collectively limit direct inter-study comparisons and extrapolation of findings. Consequently, future preclinical research priorities should include: (1) Standardized Reporting: Mandating detailed description of fucoidan’s key chemical parameters (especially MW) and animal experiment randomization/blinding procedures; (2) Improved Models: Greater use of models simulating the chronic progression of human CKD; (3) Elucidation of Dose-Response Relationships: Fundamental for confirming efficacy and safety.

**TABLE 1:** Representative preclinical studies on the protective effects of fucoidan in kidney diseases.

Source	Fraction (molecular weight)	Disease type	Experimental model	Effective dose	Exposure time	Kidney therapy	Effects on renal function	Improvement in pathology	Mechanisms	Ref.
*Saccharina japonica*	Fucoidan (220–260 kDa)	Hyperuricemia	C57BL/6J mice (n = 10)	150, 200, 300 mg/kg/d	10 weeks	Allopurinol	↓ SUA, SCr, BUN	↓ Tubular dilation, hyaline casts, glomerular atrophy, foot process fusion, apoptosis	1. ↓ Inflammation: ↓NF-κB/NLRP3; ↓ TNF-α, IL-18, IL-6, IL-1β. 2. Anti-apoptosis: ↑ Bcl-2, ↓ Bax, ↓ caspase-3. 3. ↑ Uric acid excretion: ↓ URAT1, GLUT9. 4. Modulated gut-kidney axis: ↑ gut barrier fx; modulated microbiota (↓ Blautia, Muribaculaceae, Dubosiella; ↑ *Lactobacillus*); ↑ SCFAs; ↑ intestinal ABCG2	Xue et al. (2024)
*Saccharina japonica*	Fucoidan (251.2 kDa)	Hyperuricemia-Induced fibrosis	C57BL/6J mice (n = 10)	150, 300 mg/kg/d	10 weeks	Allopurinol	↓ SUA, BUN	Improved glomerular and tubular injury; ↓ fibrosis, ECM deposition	1. ↓ Pro-fibrotic signaling: ↓JAK2/STAT3. 2. ↓ EMT: ↓ α-SMA, Col I; ↑ E-cadherin	Sun et al. (2023)
*Saccharina japonica*	LMWF(14.6 kDa)	DKD	SD rats (n = 10) HK-2 human renal proximal tubular cells	50, 100, 200 mg/kg/d 320, 640, 1,280 μg/mL	13weeks	Fosinopril	↓ MDA, GSSG; ↑ GSH, GSH/GSSG ratio	↓ Inflammation, mesangial proliferation, vacuolation, collagen deposition	1. ↑ Protective signaling: Activated Nrf-2/Keap-1/HO-1/NQO1.2. ↓ ferroptosis: Activated SLC7A11/GSH/GPX4; ↓ACSL4/FTH.	Chen X et al. (2025)
*Saccharina japonica*	LMWF (8.177 kDa)	DN	Wistar rats (n = 6)	100, 200 mg/kg/d	70 Days	Captopril	↓ Urinary micro total protein, urinary albumin, urine volume	↓ Inflammation	1. ↓ P-selectin. 2. ↓ Inflammatory cytokines: ↓ ICAM-1, IL-6, TGF-β, TNF-α, JNK, MAPK.	Xu et al. (2016)
*Saccharina japonica*	LMWF(8.84 kDa)	DKD	SD rats (n = 10)	200 mg/kg/d	13 weeks	Fosinopril	↓ BUN, SCr, urinary protein, protein/SCr ratio	↓ mesangial matrix hyperplasia, glomerular hypertrophy, tubular dilation, collagen, fibrosis	1. Regulated lipid metabolism: ↓ LXR-α, ApoE, CD36. 2. ↓ fibrotic signaling: ↓ Col I, α-SMA, Fn	Wang et al. (2021)
*Saccharina japonica*	LMWF(Not specified)	DKD	SD rats (n = 10)	50, 100, 200 mg/kg	13 weeks	Fosinopril	↓ Urinary ALB, BUN, Scr	↓ Inflammatory infiltration, collagen deposition, lipid accumulation	1.↓ Pro-lipogenic signaling: ↓ AhR/CD36 pathway. 2. ↓ Oxidative stress: ↓ ROS production	Xue et al. (2025)
*Saccharina japonica*	Fucoidan (87 kDa)	DKD	Wistar rats (n = 12)	75,150,300 mg/kg	10 weeks	Gliguidone + Lotensin	↓ Blood glucose, BUN, urine protein; ↑ Ucr and Ccr	↓ Glomerular hypertrophy, tubular degeneration	1. Modulated metabolic abnormalities: ↓ blood glucose levels. 2. Antioxidant activity: ↑ antioxidant enzymes and ↓ oxidative stress	Wang et al. (2014)
*Saccharina japonica*	Fucoidan (242 kDa)	CKD	ICR mice (n = 15)	100, 200 mg/kg	5weeks	Losartan	↓ Urinary urea, uric acid, SCr	↓ Tubular necrosis, inflammation	1. ↓ Oxidative stress: ↓GSK3β; activated Nrf2-HO-1. 2. Anti-inflammatory: ↓M1 polarization (↓ TNF-α, IL-1β, iNOS); promoted M2 polarization (↑ IL-4, TGFβ, Arg1, CD206)	Ma et al. (2022)
*Saccharina japonica*	LMWF(7.774 kDa)	DN	HRMCs	10, 20, 40, 80 μg/mL	48 h	-	-	↓ Abnormal proliferation and hypertrophy	1. Bound fibronectin: Direct interaction with FN, ↓ ECM-receptor interaction.2. ↓PI3K/AKT, JAK-STATnSignaling pathways	Wang et al. (2020)
*Saccharina japonica*	Fucoidan (136 kDa) LMWF(9.5 kDa)	NS	SD rats (n = 10)	Fucoidan: 100 mg/kg/d LMWF: 25, 50, 100 mg/kg/d	30 days	Dexamethasone	↓ Proteinuria, BUN, and Scr	-	Antioxidant: ↑SOD, ↓ MDA	Tan et al. (2020)
*Saccharina japonica*	Fucoidan (189 kDa)	AHN	Wistar rats (n = 10 or 9)	50, 100, 200 mg/kg/d	4 weeks	Dexamethasone	↓ Urinary protein excretion; ↓ plasma Cr	Restored glomerular charge selectivity	1.Improved renal hemodynamics: ↑ renal blood flow 2.Restored glomerular charge selectivity: Normalized electronegative charges in the GBM.	Zhang et al. (2005)
Not specified (Commercial product: Endocalyx™)	Fucoidan (Not specified)	CKD	C57BL/6J mice (n = 4) EA.hy926 human endothelial cells; HUVEC human umbilical vein endothelial cells	ECX 74 mg/kg/d (diet) ECX 1:1,000 dilution	2 weeks 60 min	-	-	Preserved eGC thickness; ↓ PBR	Ameliorated endothelial glycocalyx damage:1. Activated protective signaling: ERK/MAPK, PI3K. 2. Promoted exocytosis. 3. ↓enzymatic degradation	Regier et al. (2022)

Arrows indicate the direction of change:upward arrows (↑) represent activation, increase,or enhancement,and downward arrows (↓) represent inhibition, decrease,or reduction.

Abbreviations: ABCG2, ATP-binding cassette sub-family G member 2; AHN, Active Heymann nephritis; AhR,aryl hydrocarbon receptor; ALB, albumin; Arg1,arginase 1; α-SMA, alpha-smooth muscle actin; Bax, BCL2-associated X protein; Bcl-2,B-cell lymphoma 2; BUN, blood urea nitrogen; Ccr,creatinine clearance rate; CD36,cluster of differentiation 36; CD206, macrophage mannose receptor; CKD, chronic kidney disease; Col I, collagen type I; Cr, creatinine; DKD, diabetic kidney disease; DN, diabetic nephropathy; ECM, extracellular matrix; ECX, Endocalyx™; eGC, endothelial glycocalyx; EMT, epithelial-mesenchymal transition; ERK, extracellular signal-regulated kinase; Fn, fibronectin; FTH, ferritin heavy chain; Fx,function; GBM, glomerular basement membrane; GLUT9,glucose transporter 9; GPX4,glutathione peroxidase 4; GSH, glutathione; GSK3β, lycogen synthase kinase 3 beta; GSSG, glutathione disulfide; HO-1, heme oxygenase-1; HRMCs, human renal mesangial cells; HUVEC, human umbilical vein endothelial cells; ICAM-1, intercellular adhesion molecule 1; IL-1β,interleukin-1, beta; IL-18, interleukin-18; IL-4, interleukin-4; IL-6, interleukin-6; iNOS, inducible nitric oxide synthase; JAK2, Janus kinase 2; JNK, c-Jun N-terminal kinase; Keap1,Kelch-like ECH-associated protein 1; LMWF, low molecular weight fucoidan; LXR-α, liver X receptor alpha; MAPK, mitogen-activated protein kinase; MDA, malondialdehyde; NF-κB, nuclear factor kappa B; NLRP3,NLR, family pyrin domain containing 3; NQO1,NAD(P)H quinone dehydrogenase 1; Nrf2, nuclear factor erythroid 2–related factor 2; NS, nephrotic syndrome; PBR, perfused boundary region; PI3K, phosphoinositide 3-kinase; SCFAs, short-chain fatty acids; SCr, serum creatinine; SD, rats,Sprague-Dawley rats; SLC7A11, solute carrier family 7 member 11; SOD, superoxide dismutase; SPS, sulfated polysaccharides; STAT3, signal transducer and activator of transcription 3; SUA, serum uric acid; TGF-β, transforming growth factor beta; TNF-α, tumor necrosis factor alpha; Ucr, urinary creatinine; URAT1,urate anion transporter 1.

Despite these limitations, the efficacy of fucoidan has translated into clinical benefit. Drugs primarily containing fucoidan (e.g., Haikun Shenxi Capsule) have shown significant efficacy in multiple clinical studies (including randomized controlled trials and observational studies) targeting chronic renal insufficiency (Yu, 2025; Sun et al., 2025), chronic renal failure (Zhang, 2023; Cai et al., 2024; Zhang, 2024; Liu et al., 2023), diabetic kidney disease (from early stages to dialysis) (Zhang, 2025; Zhang et al., 2025; Liu et al., 2023; Shi et al., 2023; Dong and Li, 2022; Dong and Qin, 2023; Liu, 2023), and chronic glomerulonephritis (Zhu, 2023; Liu, 2022), whether used alone or in combination with conventional Western medicines (e.g., renin-angiotensin system inhibitors, liraglutide). The clinically observed significant reduction in proteinuria (manifested as decreased 24-h urine protein or UACR) is key indirect evidence of improved endothelial charge barrier and structural function. Simultaneously, the downregulation of circulating inflammatory markers (e.g., C-reactive protein, IL-6, TNF-α) reflects reduced endothelial activation and systemic inflammation. Multiple studies consistently report decreased serum creatinine and blood urea nitrogen levels, as well as stabilization or improvement in eGFR or creatinine clearance, directly demonstrating improved overall renal function and delayed CKD progression.

Regarding safety, existing clinical studies (mostly weeks to months) report adverse events primarily as mild, reversible gastrointestinal discomfort, with incidence rates not significantly different from conventional treatment groups, indicating good short-term tolerability. However, the long-term (years) safety of fucoidan (especially oral LMWF products) requires confirmation via longer follow-up studies. Particular attention should be paid to its inherent, heparin-like anticoagulant and antiplatelet activity (Chen X. et al., 2025): in advanced CKD patients who often have coagulation abnormalities and may be on antithrombotic agents, long-term supplementation could interfere with coagulation balance and increase bleeding risk. Current human studies have not identified clear negative clinical consequences on immune surveillance, extracellular matrix turnover, or vascular remodeling, but these theoretical possibilities warrant monitoring in long-term studies.

In summary, existing clinical evidence provides real-world support for fucoidan’s renoprotective effects via endothelial protection mechanisms. However, current evidence is largely based on specific compound formulations and Chinese populations. Future international, multicenter, large-scale, long-term follow-up randomized controlled trials are needed to validate the universal efficacy and long-term safety of fucoidan from different sources. Such studies should systematically incorporate safety endpoints including coagulation parameters and bleeding events and strive to integrate more direct methods for assessing endothelial function and glycocalyx status to further consolidate its clinical application.

## 6 Discussion and future perspectives

This review establishes that targeting, protecting, and repairing the endothelial glycocalyx is the central hub mechanism through which fucoidan exerts its renoprotective effects. This mechanism tightly links structural repair with functional improvement, providing a clear logic for pathological intervention. Looking forward, in-depth research and clinical translation in this field should focus on several key levels. First, regarding the mechanism of action, advanced techniques such as surface plasmon resonance (SPR) (Wang et al., 2020), isothermal titration calorimetry (ITC), and cell-specific gene editing should be employed to precisely quantify the interaction between fucoidan and glycocalyx components and validate its key cellular targets *in vivo*. Concurrently, structure-activity relationships need systematic elucidation to guide the rational design of next-generation, highly effective and specific derivatives. Second, in clinical translation, given that current clinical evidence mainly originates from specific formulations and populations, there is an urgent need for international, multicenter, large-scale, long-term follow-up randomized controlled trials to validate its efficacy and safety globally and explore its synergistic potential with existing standard therapies (e.g., sodium-glucose cotransporter-2 inhibitors). Finally, in the diagnostic and therapeutic paradigm, the endothelial glycocalyx itself shows great potential as a bridge connecting mechanism and clinical phenotype: circulating glycocalyx components could serve as dynamic biomarkers for early risk stratification, monitoring disease activity, and acting as “pharmacodynamic biomarkers” for intervention efficacy. Although establishing them as formal surrogate endpoints requires more prospective evidence, this undoubtedly represents a highly promising research direction. In conclusion, fucoidan represents a treatment candidate rooted in tradition with a novel mechanism. Through prudent scientific investigation and rigorous clinical validation, it holds promise as an important complementary strategy for the comprehensive management of CKD.
