# Supplementary material for: Fucoidan as a renal protectant: mechanistic insights and therapeutic implications of endothelial glycocalyx targeting
Source: Front Pharmacol. 2026 Jan 16;17:1749109. doi: 10.3389/fphar.2026.1749109 (PMC12855530; doi:10.3389/fphar.2026.1749109)
Supplement: Supplementary file 1 [file Table1.docx]

**Supplementary Material 1：**

**Literature Search Strategy and Study Selection Criteria**

1. Literature Search Strategy

A comprehensive literature search was conducted from inception until November 1, 2025 in the following electronic databases: PubMed, Web of Science (Core Collection), Embase, Cochrane Central Register of Controlled Trials (CENTRAL), China National Knowledge Infrastructure (CNKI), Wanfang Data, and China Biology Medicine disc (CBM). The search strategy was designed to combine Medical Subject Headings (MeSH) or equivalent controlled vocabulary terms with free-text words in the titles and abstracts. No language restrictions were applied initially, but only studies published in Chinese or English were considered for inclusion.

Example search strategy for PubMed:

("Fucoidan"[Mesh] OR fucoidan*[tiab] OR "sulfated polysaccharide"[tiab] OR "algal polysaccharide"[tiab] OR "oligo-fucoidan"[tiab] OR "low molecular weight fucoidan"[tiab] OR "LMWF"[tiab] OR "haikun shenxi"[tiab] ) AND ("Renal Insufficiency, Chronic"[Mesh] OR "Diabetic Nephropathies"[Mesh] OR "Acute Kidney Injury"[Mesh] OR "Kidney Diseases"[Mesh] OR chronic kidney disease[tiab] OR CKD[tiab] OR diabetic nephropathy[tiab] OR "renal fibrosis"[tiab] OR "kidney injury"[tiab] OR proteinuria[tiab] OR albuminuria[tiab] OR "renal protect*"[tiab]) AND ("Endothelial Glycocalyx"[Mesh] OR endothelial glycocalyx[tiab] OR glycocalyx[tiab] OR "heparan sulfate"[Mesh] OR heparan sulfate[tiab] OR "Syndecan-1"[tiab] OR "Syndecan"[tiab] OR "Heparanase"[Mesh] OR heparanase[tiab] OR "HPSE"[tiab] OR "charge barrier"[tiab] OR "glomerular filtration barrier"[tiab] OR "vascular permeability"[tiab]).

Example search strategy for CNKI (Chinese search formula example):

((SU%='海昆肾喜' OR TI='海昆肾喜' OR KY='海昆肾喜' OR AB='海昆肾喜' OR SU%='褐藻多糖硫酸酯胶囊' OR TI='褐藻多糖硫酸酯胶囊' OR AB='褐藻多糖硫酸酯胶囊' OR TI='Haikunshenxi' OR AB='Haikunshenxi') OR (SU%='岩藻多糖' OR TI='岩藻多糖' OR KY='岩藻多糖' OR AB='岩藻多糖' OR SU%='褐藻多糖硫酸酯' OR TI='褐藻多糖硫酸酯' OR AB='褐藻多糖硫酸酯' OR SU%='硫酸化多糖' OR AB='硫酸化多糖' OR TI='海藻多糖' OR AB='海藻多糖' OR TI='低分子岩藻多糖' OR AB='低分子岩藻多糖' OR AB='低分子量岩藻多糖' OR TI='LMWF' OR AB='LMWF')) AND (SU%='慢性肾脏病' OR SU%='慢性肾病' OR TI='慢性肾病' OR AB='慢性肾病' OR SU%='糖尿病肾病' OR TI='糖尿病肾病' OR AB='糖尿病肾病' OR SU%='急性肾损伤' OR TI='急性肾损伤' OR AB='急性肾损伤' OR SU%='肾脏疾病' OR TI='肾纤维化' OR AB='肾纤维化' OR TI='肾损伤' OR AB='肾损伤' OR TI='蛋白尿' OR AB='蛋白尿' OR TI='白蛋白尿' OR AB='白蛋白尿' OR AB='肾脏保护' OR TI='肾脏保护') AND (SU%='内皮糖萼' OR TI='内皮糖萼' OR AB='内皮糖萼' OR SU%='糖萼' OR TI='糖萼' OR AB='糖萼' OR SU%='硫酸乙酰肝素' OR TI='硫酸乙酰肝素' OR AB='硫酸乙酰肝素' OR TI='多配体蛋白聚糖-1' OR AB='多配体蛋白聚糖-1' OR TI='Syndecan-1' OR AB='Syndecan-1' OR TI='乙酰肝素酶' OR AB='乙酰肝素酶' OR TI='HPSE' OR AB='HPSE' OR AB='电荷屏障' OR AB='肾小球滤过屏障' OR AB='血管通透性').

2.Literature Screening and Synthesis Approach

Literature screening was conducted independently by two investigators (Ping Xin and Chengqiao Ge). Initially, titles and abstracts were screened to exclude obviously irrelevant studies (e.g., those focusing on non-renal diseases). Subsequently, full texts of potentially eligible articles were reviewed for final judgment based on the pre-defined inclusion and exclusion criteria. For evidence synthesis, we prioritized studies with clear mechanistic insights and robust experimental design. Data specifically pertaining to the effects of fucoidan on glycocalyx components (e.g., heparan sulfate, syndecan-1) and the associated downstream renoprotective outcomes were extracted and synthesized to construct the core narrative of this review.

3. Study Selection Criteria

Inclusion Criteria:

(i) Original research studies (in vitro, animal, or clinical investigations) examining fucoidan and/or its preparation "Haikun Shenxi";

(ii) Studies focusing on kidney diseases and/or the endothelial glycocalyx;

(iii) Studies that directly investigated or discussed the mechanisms related to the modulation or repair of the glycocalyx by fucoidan.

Exclusion Criteria:

(i) Studies unrelated to fucoidan, kidney diseases, or the glycocalyx;

(ii) Conference abstracts, commentaries, editorials, and other non-research publications;

(iii) Studies focusing on other diseases without assessment of kidney-related outcomes or glycocalyx status;

(iv) Duplicate publications.
