# Supplementary material for: Fucoidan as a renal protectant: mechanistic insights and therapeutic implications of endothelial glycocalyx targeting
Source: Front Pharmacol. 2026 Jan 16;17:1749109. doi: 10.3389/fphar.2026.1749109 (PMC12855530; doi:10.3389/fphar.2026.1749109)
Supplement: Supplementary file 2 [file Table2.docx]

## Supplementary Material 2

Table 2 Summary of Some Clinical Research Results of HaiKun ShenXi Capsules / Fucoidan in the Treatment of Kidney Diseases (in the Past 3 Years)

| Study Type | Disease | Sample Size (Experimental/Control) | Intervention | Control | Duration | Outcomes | Adverse Reactions | REF. |
| --- | --- | --- | --- | --- | --- | --- | --- | --- |
| RCT | Chronic Renal Failure | 53 (27/26) | Shenkang Injection + Haikun Shenxi Capsule | Shenkang Injection | 1 month | ↑ Clinical total effective rate; ↓ Symptom score; ↓ BUN, UA, Scr, β2-MG | No difference in adverse reactions (Exp: diarrhea, nausea 1 case each; Con: diarrhea, nausea, headache, abdominal distension 1 case each) | Zhang et al. (2023) |
| Observational | CKD Stages 2-5 | 120 (50/39/31) | C: Chinese Herbal Decoction + Haikun Shenxi Capsule | A: Haikun Shenxi Capsule; B: Chinese Herbal Decoction | 4 weeks | ↓ Scr; ↑ eGFR; ↓ TCM syndrome score; ↑ Clinical total effective rate | Not mentioned | Sun et al. (2025) |
| RCT | Chronic Renal Insufficiency | 90 (45/45) | Obs: Niaoduqing + Haikun Shenxi Capsule | Niaoduqing | 28 days | ↑ Total treatment effective rate; Improved symptom relief | Not mentioned | Yu et al. (2025) |
| RCT | Diabetic Kidney Disease | 107 (54/53) | Obs: Liraglutide + Pancreatic Kininogenase + Haikun Shenxi Capsule | Liraglutide + Pancreatic Kininogenase | 6 months | ↑ Total treatment effective rate; Improved glycemic variability; ↓ BUN, Scr, 24h Upro; ↑ eGFR; ↓ IL-23, IL-17; ↓ Urinary podocyte markers | No difference in adverse reactions (Obs: nausea, vomiting, fatigue 1 case each; Con: nausea, fatigue 2 cases each, vomiting 1 case) | Zhang et al. (2025) |
| RCT | Elderly Diabetic Nephropathy | 80 (40/40) | Exp: Irbesartan + Haikun Shenxi Capsule | Irbesartan | 2 months | Improved blood glucose levels; Improved vascular endothelial function; ↓ Clinical symptom score; Improved renal function indicators | Not mentioned | Zhang et al. (2025) |
| RCT | Chronic Renal Failure | 98 (49/49) | Obs: Chinese Herbal Decoction + Haikun Shenxi Capsule | Haikun Shenxi Capsule | 1 month | ↑ Therapeutic effect; ↓ TCM syndrome score; ↓ BUN, Scr, 24h Upro; Improved oxidative stress and inflammatory factors | No difference in adverse reactions (Obs: gastric discomfort 1 case, poor appetite 2 cases, nausea/vomiting 1 case, indigestion 1 case; Con: gastric discomfort 1 case, poor appetite 1 case, nausea/vomiting 1 case, indigestion 2 cases) | Cai et al. (2024) |
| RCT | Chronic Renal Failure | 92 (46/46) | Haikun Shenxi Capsule + L-Carnitine | L-Carnitine | 2 months | ↓ BUN, Scr, Cys-C; ↑ CCr; ↓ hs-CRP, IL-6, IL-8 | No difference in adverse reactions (Obs: indigestion 2 cases, poor appetite 1 case, nausea 2 cases; Con: poor appetite, nausea, diarrhea 1 case each) | Zhang et al. (2024) |
| RCT | Chronic Glomerulonephritis | 186 (93/93) | Haikun Shenxi Capsule + Valsartan | Valsartan | 3 months | ↑ Total treatment effective rate; ↑ GFR; ↓ UA, Cys-C; ↓ NF-κB, VEGF, sFlt-1 | No difference in adverse reactions (Obs: nausea/vomiting 4 cases, diarrhea/abdominal pain 2 cases, dizziness/headache 5 cases; Con: nausea/vomiting 6 cases, diarrhea/abdominal pain 6 cases, dizziness/headache 7 cases) | Zhu et al. (2023) |
| RCT | Chronic Renal Failure | 76 (38/38) | Chinese Herbal Decoction + Haikun Shenxi Capsule | Haikun Shenxi Capsule | 2 months | ↑ Treatment effective rate; Improved renal function (Scr, BUN, 24h Upro); Improved oxidative stress markers (SOD, MDA, AOPP) | Not mentioned | Liu Y et al. (2023) |
| RCT | Diabetic Nephropathy (Hemodialysis) | 103 (52/51) | Haikun Shenxi Capsule + Benazepril | Benazepril | 4 weeks | ↑ Clinical efficacy; ↓ Scr, BUN, 24h UP, CysC; ↓ TNF-α, IL-6, CRP | No difference in adverse reactions (Obs: hypotension 1 case, GI reaction 3 cases, fatigue 2 cases; Con: hypotension 2 cases, GI reaction 2 cases) | Liu F et al. (2023) |
| RCT | Elderly Early Diabetic Nephropathy | 84 (42/42) | Exp: Haikun Shenxi Capsule + Valsartan | Valsartan | 3 months | ↑ Total treatment effective rate; ↓ hs-CRP, TNF-α; ↓ TGF-β1, MMP-2; Improved renal function $\text{BUN}\text{,}\text{Cr}\text{,}\text{β}\text{2−}\text{MG}\text{,24h}\text{Upro}$ | Not mentioned | Shi et al. (2023) |
| RCT | Diabetic Nephropathy with Chronic Renal Insufficiency | 240 (120/120) | Obs: Calcium Dobesilate + Haikun Shenxi Capsule | Calcium Dobesilate | 2 months | ↓ BUN, Scr, UAER; ↑ Ccr; ↓ TCM syndrome score; ↑ Total effective rate | No difference in adverse reactions (Obs: GI discomfort 1 case; Con: GI discomfort 3 cases) | Dong et al. (2023) |
| RCT | Chronic Renal Failure | 80 (40/40) | Obs: Haikun Shenxi Capsule + Benazepril | Benazepril | 1 month | ↑ Clinical total effective rate; ↓ TCM syndrome score; Improved oxidative stress (SOD, MDA, AOPP, AGEs); Improved renal function (BUN, Scr, Ccr) | No difference in adverse reactions (Obs: headache 1 case, abdominal distension 1 case, nausea 1 case; Con: headache 2 cases, nausea 1 case, drowsiness 2 cases, fatigue 1 case) | Ying et al. (2023) |

Abbreviations: ↑: Increased; ↓: Decreased, BUN: Blood Urea Nitrogen, UA: Uric Acid, Scr: Serum Creatinine, β2-MG: β2-microglobulin, eGFR: estimated Glomerular Filtration Rate, TCM: Traditional Chinese Medicine, Upro: Urinary Protein, Ccr: Creatinine Clearance Rate, Cys-C: Cystatin C, hs-CRP: high-sensitivity C-Reactive Protein, IL: Interleukin, TNF-α: Tumor Necrosis Factor-alpha, NF-κB: Nuclear Factor Kappa B, VEGF: Vascular Endothelial Growth Factor, sFlt-1: soluble Fms-like Tyrosine Kinase-1, SOD: Superoxide Dismutase, MDA: Malondialdehyde, AOPP: Advanced Oxidation Protein Products, AGEs: Advanced Glycation End products, UAER: Urinary Albumin Excretion Rate, UPQ: Urinary Protein Quantification, GI: Gastrointestinal, RCT: Randomized Controlled Trial, CKD: Chronic Kidney Disease, Exp: Experimental Group, Obs: Observation Group, Con: Control Group
